# Incursions of *Candida auris* into Australia, 2018

**DOI:** 10.3201/eid2606.190936

**Published:** 2020-06

**Authors:** Courtney R. Lane, Torsten Seemann, Leon J. Worth, Marion Easton, William Pitchers, Jenny Wong, Donna Cameron, Francesca Azzato, Richard Bartolo, Cristina Mateevici, Caroline Marshall, Monica A. Slavin, Benjamin P. Howden, Deborah A. Williamson

**Affiliations:** The University of Melbourne at the Peter Doherty Institute for Infection and Immunity, Melbourne, Victoria, Australia (C.R. Lane, T. Seemann, W. Pitchers, D. Cameron, B.P. Howden, D.A. Williamson);; VICNISS Coordinating Centre at the Peter Doherty Institute for Infection and Immunity, Melbourne (L.J. Worth);; University of Melbourne, Parkville, Victoria, Australia (L.J. Worth, C. Marshall, M.A. Slavin);; Peter MacCallum Cancer Centre, Melbourne (L.J. Worth, M.A. Slavin);; Victorian Department of Health and Human Services, Melbourne (M. Easton, D. Cameron);; Dorevitch Pathology—Western Health, Footscray, Victoria (J. Wong);; Royal Melbourne Hospital at the Peter Doherty Institute for Infection and Immunity, Melbourne (F. Azzato, C. Marshall, M.A. Slavin);; Western Health, Footscray (R. Bartolo, C. Mateevici);; Royal Melbourne Hospital, Parkville (C. Marshall, D.A. Williamson);; Austin Health, Heidelberg, Victoria, Australia (B.P. Howden)

**Keywords:** antimicrobial resistance, public health surveillance, *Candida auris*, infection control, genomics, fungi, Australia

## Abstract

*Candida auris* is an emerging global healthcare-associated pathogen. During July–December 2018, four patients with *C. auris* were identified in Victoria, Australia, all with previous overseas hospitalization. Phylogenetic analysis revealed putative transmission between 2 patients and suspected overseas acquisition in the others. Vigilant screening of at-risk patients is required.

The fungal pathogen *Candida auris* is an emerging global health threat associated with a range of invasive infections, most commonly candidemia; it is often resistant to multiple antifungal drugs (*1*). First identified in Japan in 2009, *C. auris* has been reported across all 6 populated continents with outbreaks in healthcare settings, particularly intensive care and high-dependence units (*1*,*2*). Four genetic lineages of *C. auris* with phylogeographic variation have been identified (*3*).

Before July 2018, only 1 case of *C. auris* had been reported in Australia, none in the state of Victoria (population 6.5 million) (*4*); no centralized surveillance or mandatory reporting has been implemented on a state or national level, and local screening policies are limited. However, Victoria has experienced large interfacility and intrafacility healthcare-associated outbreaks of other multidrug-resistant organisms (*5*) and has increasingly implemented genomics in both the investigation of outbreaks and routine surveillance (C.R. Lane et al., unpub. data, https://papers.ssrn.com/sol3/papers.cfm?abstract_id=3498431).

In July 2018, *C. auris* was cultured from a patient hospitalized in a Victoria healthcare facility. In response, the Victoria Department of Health and Human Services (DHHS) convened an incident management team and issued a Chief Health Officer alert to all health services and laboratories recommending admission screening for patients with recent overseas hospitalization. Also recommended was consideration of *C. auris* in patients with cultured non–*C. albicans* species and risk factors for fungal infection, including diabetes mellitus and recent antimicrobial drug use. The alert specified that all *C. auris* and nonspeciated non–*C. albicans* isolates from high-risk patients be referred to Victorian Public Health laboratories for speciation and characterization and reported to the DHHS (*6*). We report on the use of genomics to investigate putative transmission of *C. auris* in Victoria during July 1–December 31, 2018.

Isolates of *C. auris* were referred to the Victorian Infectious Diseases Reference Laboratory, where they underwent species identification and antimicrobial susceptibility testing. All isolates were then referred to the Microbiological Diagnostic Unit Public Health Laboratory for DNA extraction, whole-genome sequencing, and bioinformatic analysis.

During July–December 2018, we identified *C. auris* in 4 patients. Three patients (patients 1, 2, and 4 chronologically) were identified through clinically indicated samples, whereas patient 3 was screened on transfer from an overseas healthcare facility. Patients 1 and 2 were admitted to the same hospital at specimen collection, and patients 3 and 4 were admitted to different facilities. All patients reported previous overseas hospitalization (Figure).

**Figure:**
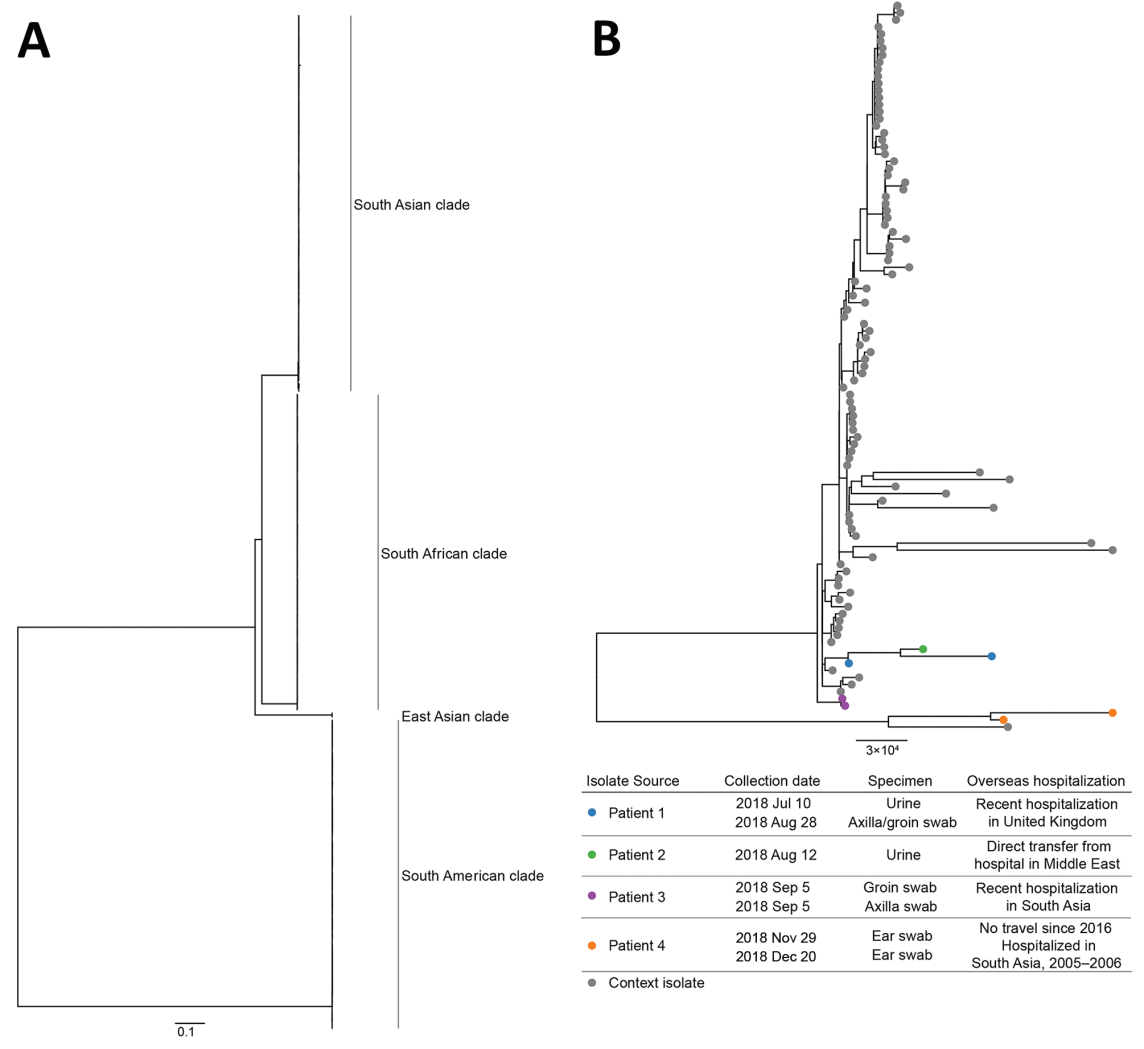
Maximum-likelihood phylogenetic trees of *Candida auris* isolates from Victoria, Australia, in the context of international publicly available genomes. A) Complete tree; B) South Asian clade. Isolates from 4 patients in Victoria are indicated by colored dots on the inset tree; isolate details and patient travel history are provided in the key. Scale bars indicate substitutions per site.

We obtained 7 *C. auris* isolates from these 4 patients and performed core genome phylogenetic analysis on all isolates (Appendix). We downloaded publicly available *C. auris* sequences and included those meeting quality control metrics (Appendix Table 1). Phylogenetic analysis revealed all 7 isolates fell within the previously described South Asian clade (Figure, panel A).

We identified putative transmission during a concurrent hospital stay between patients 1 and 2 (Figure, panel B); transmission was epidemiologically validated and reported elsewhere (*7*). Because both patients reported overseas hospitalization, it is unclear which constituted the index case. Isolates from patients 3 and 4 were not closely related to each other, or any other included isolates, consistent with independent overseas acquisition.

We reported results of phylogenetic analyses prospectively and concurrently to the incident management team and affected facilities. Because all patients reported overseas hospitalization, the combined analysis of genomic and epidemiologic data enabled assessment of alternative hypotheses, identifying putative local transmission between patients 1 and 2 and excluding patients 3 and 4 from the outbreak. These results justified targeted infection control measures.

In late 2015, Victoria introduced combined phylogenetic and epidemiologic surveillance for carbapenemase-producing *Enterobacterales*, a similar low-prevalence multiresistant organism (*8*). Using a search-and-destroy approach, this method enabled early identification of local transmission and complemented standard laboratory, screening, and outbreak control measures across the state. Leveraging from this work, a similar system was introduced for the control of *C. auris* in September 2019, with statewide mandatory notification of *C. auris* introduced in December 2019 (*9**,**10*). These measures address limitations in the current study, such as the inability to identify patients with *C. auris* because of noncompliance with screening recommendations, nonreporting, or decreased sensitivity of laboratory methods for the detection of *C. auris*.

A representative sample of international isolates enables inference of local transmission through local cluster identification and can indicate the source of local strains. The emergence of *C. auris* highlights the need for greater surveillance of nonbacterial multiresistant organisms and international data sharing. Sequences from our study were submitted to GenBank.

Our findings demonstrate the importance of proactive screening programs and of strict isolation and containment actions. Despite Australia’s geographic isolation, vigilance is necessary to ensure that patients hospitalized overseas are identified and screened for the presence of multiresistant organisms such as *C. auris* upon hospital admission.

Appendix Additional information about *Candida auris* in Australia.
