# Supplementary material for: Incursions of Candida auris into Australia, 2018
Source: Emerg Infect Dis. 2020 Jun;26(6):1326–8. doi: 10.3201/eid2606.190936 (PMC7258446; doi:10.3201/eid2606.190936)
Supplement: Appendix — Additional information about Candida auris in Australia. [file 19-0936-Techapp-s1.pdf]

Article DOI: https://doi.org/10.3201/eid2606.190936

Incursions of *Candida auris* into Australia, 2018

Appendix

Methods

Identification and Antifungal Susceptibility Testing

We confirmed isolates as *C. auris* by matrix-assisted laser desorption/ionization time-of-flight mass spectrometry (MALDI-TOF; Bruker Biotyper, https://www.bruker.com), and performed antifungal susceptibility testing by broth microdilution (Sensititre YeastOne, https://www.thermofisher.com).

DNA Extraction and Whole-Genome Sequencing

We performed DNA extraction and whole-genome sequencing (WGS) of study isolates at the Microbiological Diagnostic Unit Public Health Laboratory at the University of Melbourne. Genomic DNA was extracted from a single colony using a QIAsymphony DSP DNA Mini Kit (QIAGEN, https://www.qiagen.com) according to manufacturer's instructions, and WGS was performed on an Illumina NextSeq 500 platform (Illumina, https://www.illumina.com) with 150 bp paired-end reads or Illumina MiSeq platform with 75 bp paired-end reads. We resequenced isolates that had sequencing depth $\geq$50× and a minimum Phred quality score of 30. Reads are available from the NCBI Sequence Read Archive (BioProject, https://www.ncbi.nlm.nih.gov/bioproject).

All *C. auris* sequences available on public databases were downloaded and assessed against the quality metrics above; we excluded sequences not meeting these targets.

SNP and Phylogenetic Analysis

We initially compared study genomes and publicly available genomes with *Candida auris* strain B8441 using Snippy version 4.3 (https://github.com/tseemann/snippy). We performed alignment using BWA MEM version 0.7.17-r1188 (1) and called single nucleotide polymorphisms (SNPs) using Freebayes version 1.2.0 (E. Garrison et al., unpub.data,

https://arxiv.org/abs/1207.3907), requiring a minimum read coverage of 10, minimum base quality of 13 and 90% read concordance at a site to report SNPs. The resulting core SNP alignment consisted of 151,484 sites. We inferred maximum likelihood (ML) phylogenetic trees using RAxML version 8.2.12 with 100 pseudo-bootstrap replicates and using a generalized time-reversible model, a Gamma distribution to model site-specific rate variation (the GTR + Γ substitution mode: GTRGAMMA in RAxML).

We performed subanalysis of the identified South Asian clade, including all locally sequenced isolates, using the methods above. The resulting core SNP alignment consisted of 103 sites.

We filtered these alignments for phage regions identified using Phaster (2) and recombination regions identified using Gubbins version 2.3.4 (*3*), and extracted core SNPs using SNP-sites (*4*). The resulting trees were midpoint-rooted with *phangorn* (v2.3.1) (*5*) and rendered using *ggtree* (1.8.1) (*6*). We calculated pairwise SNP distances using *harrietR* (https://github.com/andersgs/harrietr).

Appendix Table 1. Publicly available *Candida auris* isolates included in phylogenetic analysis, Victoria, Australia

Accession number	Year	Country	Accession no.	Year	Country
ERR2299885	Unknown	United Kingdom	ERR2300789	Unknown	Unknown
ERR2299874	Unknown	United Kingdom	ERR2300809	Unknown	Unknown
ERR2299868	Unknown	United Kingdom	ERR2300771	Unknown	Unknown
ERR2299870	Unknown	United Kingdom	ERR2300797	Unknown	Unknown
ERR2299877	Unknown	United Kingdom	ERR2300772	Unknown	Unknown
ERR2299887	Unknown	United Kingdom	ERR2300796	Unknown	Unknown
ERR2299892	Unknown	United Kingdom	SRR7976616	2015	United Kingdom
ERR2299893	Unknown	United Kingdom	SRR7976613	2017	United Kingdom
ERR2299891	Unknown	United Kingdom	SRR7976550	2016	United Kingdom
ERR2299882	Unknown	United Kingdom	SRR7976606	2017	United Kingdom
ERR2299884	Unknown	United Kingdom	SRR7976580	2017	United Kingdom
ERR2299881	Unknown	United Kingdom	SRR7976597	2016	United Kingdom
ERR2299872	Unknown	United Kingdom	SRR7976581	2017	United Kingdom
ERR2299883	Unknown	United Kingdom	SRR7976611	2017	United Kingdom
ERR2299871	Unknown	United Kingdom	SRR7976549	2015	United Kingdom
ERR2299869	Unknown	United Kingdom	SRR7976604	2017	United Kingdom
ERR2299873	Unknown	United Kingdom	SRR7976593	2017	United Kingdom
ERR2299875	Unknown	United Kingdom	SRR7976569	2017	United Kingdom
ERR2299886	Unknown	United Kingdom	SRR7976603	2017	United Kingdom
ERR2299880	Unknown	United Kingdom	SRR7976614	2016	United Kingdom
ERR2299878	Unknown	United Kingdom	SRR7976579	2015	United Kingdom
ERR2299889	Unknown	United Kingdom	SRR7976570	2017	United Kingdom
ERR2299890	Unknown	United Kingdom	SRR7976558	2017	United Kingdom
ERR2299876	Unknown	United Kingdom	SRR7976599	2017	United Kingdom
ERR2299888	Unknown	United Kingdom	SRR7976556	2017	United Kingdom
SRR3883466	2012	Venezuela	SRR7976594	2017	United Kingdom
SRR3883468	2012	Venezuela	SRR7976562	2017	United Kingdom
SRR3883464	2013	Venezuela	SRR7976565	2017	United Kingdom
SRR3883465	2012	Venezuela	SRR7976575	2017	United Kingdom
SRR3883467	2012	Venezuela	SRR7976560	2017	United Kingdom
SRR7140043	2016	Colombia	SRR7976589	2017	United Kingdom
SRR7140042	2016	Colombia	SRR7976577	2016	United Kingdom
SRR7140052	2016	Colombia	SRR7976559	2017	United Kingdom

Accession number	Year	Country	Accession no.	Year	Country
SRR7140069	2016	Colombia	SRR7976608	2017	United Kingdom
SRR7140058	2016	Colombia	SRR7976553	2017	United Kingdom
SRR7140059	2016	Colombia	SRR7976587	2017	United Kingdom
SRR7140010	2016	Colombia	SRR7976584	2016	United Kingdom
SRR7140009	2016	Colombia	SRR7976543	2017	United Kingdom
SRR7140022	2016	Colombia	SRR7976601	2017	United Kingdom
SRR7140020	2016	Colombia	SRR7976612	2017	United Kingdom
SRR7140076	2016	Colombia	SRR7976576	2017	United Kingdom
SRR7140006	2016	Colombia	SRR7976605	2017	United Kingdom
SRR7140044	2016	Colombia	SRR7976590	2017	United Kingdom
SRR7140025	2016	Colombia	SRR7976547	2017	United Kingdom
SRR7140063	2016	Colombia	SRR7976586	2017	United Kingdom
SRR7140032	2016	Colombia	SRR7976602	2017	United Kingdom
SRR7140007	2016	Colombia	ERR2300804	Unknown	Unknown
SRR7140001	2016	Colombia	ERR2300783	Unknown	Unknown
SRR7140035	2016	Colombia	SRR7976566	2017	United Kingdom
SRR7140039	2016	Colombia	SRR7976583	2017	United Kingdom
SRR7140045	2016	Colombia	SRR7976564	2017	United Kingdom
SRR7140061	2016	Colombia	SRR7976600	2017	United Kingdom
SRR7140004	2015	Colombia	SRR7976552	2017	United Kingdom
SRR7140054	2016	Colombia	SRR7976609	2017	United Kingdom
SRR7140033	2015	Colombia	SRR7976610	2017	United Kingdom
SRR7140038	2016	Colombia	SRR7976541	2017	United Kingdom
SRR7140013	2016	Colombia	SRR7976572	2017	United Kingdom
SRR7140040	2016	Colombia	SRR7976544	2017	United Kingdom
SRR7140030	2016	Colombia	SRR7976617	2017	United Kingdom
SRR7140082	2015	Colombia	SRR7976598	2017	United Kingdom
SRR7140017	2016	Colombia	SRR7976545	2017	United Kingdom
SRR7140024	2016	Colombia	SRR7976555	2017	United Kingdom
SRR7140047	2016	Colombia	SRR7976573	2017	United Kingdom
SRR7140003	2016	Colombia	SRR7976585	2017	United Kingdom
SRR7140068	2016	Colombia	SRR7976582	2015	United Kingdom
SRR7140074	2016	Colombia	SRR7976540	2017	United Kingdom
SRR7140041	2015	Colombia	SRR7976567	2017	United Kingdom
SRR7140029	2015	Colombia	SRR7976546	2017	United Kingdom
SRR7140080	2016	Colombia	SRR7976548	2017	United Kingdom
SRR7140056	2015	Colombia	SRR7976615	2017	United Kingdom
SRR7140070	2015	Colombia	SRR7976591	2016	United Kingdom
SRR7140015	2015	Colombia	SRR7976561	2017	United Kingdom
SRR7140012	2015	Colombia	SRR7976557	2016	United Kingdom
SRR7140071	2015	Colombia	SRR7976568	2017	United Kingdom
SRR7140077	2016	Colombia	SRR7976574	2017	United Kingdom
SRR7140062	2015	Colombia	SRR7976592	2017	United Kingdom
SRR7140002	2016	Colombia	SRR3883473	2015	Pakistan
SRR7140055	2015	Colombia	SRR3883439	Unknown	India
SRR7140075	2016	Colombia	SRR3883437	Unknown	India
SRR7140008	2015	Colombia	SRR3883451	Unknown	India
SRR7140078	2016	Colombia	SRR3883436	Unknown	India
SRR7140066	2016	Colombia	ERR899743	Unknown	Unknown
SRR7140065	2016	Colombia	SRR3883434	Unknown	India
SRR7140026	2016	Colombia	ERR1519358	Unknown	Unknown
SRR7140023	2016	Colombia	ERR1519357	Unknown	Unknown
SRR7140049	2016	Colombia	SRR3883435	Unknown	India
SRR7140073	2016	Colombia	SRR3883440	Unknown	India
SRR7140027	2016	Colombia	SRR3883449	2014	Pakistan
SRR7140072	2016	Colombia	SRR3883429	2015	Pakistan
SRR7140014	2016	Colombia	SRR3883471	2015	Pakistan
SRR7140037	2016	Colombia	SRR3883430	2015	Pakistan
SRR7140021	2015	Colombia	SRR3883438	2014	Pakistan
SRR7140064	2016	Colombia	ERR1519359	Unknown	Unknown
SRR7140036	2015	Colombia	SRR1664628	2013	India
SRR7140067	2015	Colombia	SRR1664626	2013	India
SRR7140016	2016	Colombia	SRR1664627	2013	India
SRR7140031	2015	Colombia	SRR3883460	2014	Pakistan
SRR7140011	2016	Colombia	SRR3883470	2014	Pakistan
SRR7140028	2016	Colombia	SRR3883426	2014	Pakistan
SRR7140034	2016	Colombia	SRR3883432	2015	Pakistan
SRR7140051	2016	Colombia	SRR3883474	2015	Pakistan
SRR7140019	2016	Colombia	SRR3883427	2014	Pakistan

Accession number	Year	Country	Accession no.	Year	Country
SRR7140048	2016	Colombia	SRR3883431	2015	Pakistan
SRR7140057	2015	Colombia	SRR3883428	2015	Pakistan
SRR7140079	2016	Colombia	SRR3883433	2015	Pakistan
SRR7140018	2016	Colombia	SRR3883444	Unknown	India
SRR7140046	2016	Colombia	ERR2300793	Unknown	Unknown
SRR7140050	2016	Colombia	ERR2300806	Unknown	Unknown
SRR7140060	2016	Colombia	ERR2300790	Unknown	Unknown
SRR7140053	2016	Colombia	ERR2300792	Unknown	Unknown
SRR7140005	2016	Colombia	ERR2300805	Unknown	Unknown
SRR7140081	2016	Colombia	ERR2300784	Unknown	Unknown
ERR2300774	Unknown	Unknown	ERR2300803	Unknown	Unknown
SRR3883452	2009	Japan	ERR2300794	Unknown	Unknown
SRR3883463	2014	South Africa	ERR2300791	Unknown	Unknown
SRR3883457	2014	South Africa	ERR2300799	Unknown	Unknown
SRR3883455	2012	South Africa	SRR3883441	Unknown	India
SRR3883453	2012	South Africa	SRR3883450	Unknown	India
SRR3883454	2012	South Africa	SRR3883446	Unknown	India
SRR3883461	2014	South Africa	SRR7507279	2017	USA
SRR3883456	2012	South Africa	SRR3883472	2015	Pakistan
SRR3883458	2014	South Africa	SRR6220384	2015	Pakistan
SRR3883459	2014	South Africa	SRR3883447	Unknown	India
SRR3883462	2014	South Africa	SRR3883442	Unknown	India
ERR2300770	Unknown	Unknown	SRR3883445	Unknown	India
ERR2300798	Unknown	Unknown	SRR3883443	Unknown	India
ERR2300810	Unknown	Unknown	SRR3883448	Unknown	India
ERR2300769	Unknown	Unknown			

Appendix Table 2. Antimicrobial susceptibility profile of isolates of *Candida auris* identified in Victoria, Australia, July– December 2018

		Specimen	Minimum inhibitory concentration (µg/mL)				
Patient	Collection date	source	Amphotericin B	Fluconazole	Voriconazole	Micofungin	Anidulafungin
1	10-Jul-18	Urine	0	256	1	0.12	0.12
2	12-Aug-18	Urine	4	256	2	0.25	0.25
3	5-Sep-18	Groin swab	2	256	1	0.12	0.12
	5-Sep-18	Axilla swab	2	256	1	0.12	0.12
4	29-Nov-18	Ear swab	0.5	16	0.5	2	0.5

References

1. Li H, Durbin R. Fast and accurate long-read alignment with Burrows-Wheeler transform. Bioinformatics. 2010;26:589–95. PubMed https://doi.org/10.1093/bioinformatics/btp698

2. Stamatakis A. RAxML version 8: a tool for phylogenetic analysis and post-analysis of large phylogenies. Bioinformatics. 2014;30:1312–3. PubMed https://doi.org/10.1093/bioinformatics/btu033

3. Page AJ, Taylor B, Delaney AJ, Soares J, Seemann T, Keane JA, et al. *SNP-sites*: rapid efficient extraction of SNPs from multi-FASTA alignments. Microb Genom. 2016;2:e000056. PubMed

4. Croucher NJ, Page AJ, Connor TR, Delaney AJ, Keane JA, Bentley SD, et al. Rapid phylogenetic analysis of large samples of recombinant bacterial whole genome sequences using Gubbins. Nucleic Acids Res. 2015;43:e15. PubMed https://doi.org/10.1093/nar/gku1196

5. Schliep KP. phangorn: phylogenetic analysis in R. Bioinformatics. 2011;27:592–3. PubMed https://doi.org/10.1093/bioinformatics/btq706

6. Yu G, Smith DK, Zhu H, Guan Y, Lam TTY. GGTREE: an R package for visualization and annotation of phylogenetic trees with their covariates and other associated data. Methods Ecol Evol. 2017;8:28–36. https://doi.org/10.1111/2041-210X.12628
